# Tumour necrosis factor-α promotes liver ischaemia-reperfusion injury through the PGC-1α/Mfn2 pathway

**DOI:** 10.1111/jcmm.12320

**Published:** 2014-06-04

**Authors:** Jun Li, Wenbo Ke, Qi Zhou, Yongzhong Wu, Hong Luo, Hong Zhou, Bin Yang, Yu Guo, Qichang Zheng, Yong Zhang

**Affiliations:** a Department of Urology Surgery, Cancer InstituteChongqing, China; b Department of Hepatobiliary Surgery, Union Hospital, Tongji Medical College, Huazhong University of Science and TechnologyWuhan, China; c Department of Gynecologic Oncology, Cancer InstituteChongqing, China; d Department of Radiotherapy, Cancer InstituteChongqing, China; e Department of Anorectal Surgery, NO.1 People's Hospital, Hubei University of MedcineXiangyang, China

**Keywords:** liver, tumour necrosis factor alpha, ischaemia-reperfusion injury, peroxisome proliferator–activated receptor-γ co-activator-1α, mitofusion2

## Abstract

Tumour necrosis factor (TNF)-α has been considered to induce ischaemia-reperfusion injury (IRI) of liver which is characterized by energy dysmetabolism. Peroxisome proliferator–activated receptor-γ co-activator (PGC)-1α and mitofusion2 (Mfn2) are reported to be involved in the regulation of mitochondrial function. However, whether PGC-1α and Mfn2 form a pathway that mediates liver IRI, and if so, what the underlying involvement is in that pathway remain unclear. In this study, L02 cells administered recombinant human TNF-α had increased TNF-α levels and resulted in down-regulation of PGC-1α and Mfn2 in a rat liver IRI model. This was associated with hepatic mitochondrial swelling, decreased adenosine triphosphate (ATP) production, and increased levels of reactive oxygen species (ROS) and alanine aminotransferase (ALT) activity as well as cell apoptosis. Inhibition of TNF-α by neutralizing antibody reversed PGC-1α and Mfn2 expression, and decreased hepatic injury and cell apoptosis both in cell culture and in animals. Treatment by rosiglitazone sustained PGC-1α and Mfn2 expression both in IR livers, and L02 cells treated with TNF-α as indicated by increased hepatic mitochondrial integrity and ATP production, reduced ROS and ALT activity as well as decreased cell apoptosis. Overexpression of Mfn2 by lentiviral-Mfn2 transfection decreased hepatic injury in IR livers and L02 cells treated with TNF-α. However, there was no up-regulation of PGC-1α. These findings suggest that PGC-1α and Mfn2 constitute a regulatory pathway, and play a critical role in TNF-α-induced hepatic IRI. Inhibition of the TNF-α or PGC-1α/Mfn2 pathways may represent novel therapeutic interventions for hepatic IRI.

## Introduction

Ischaemia-reperfusion injury (IRI) is the most important cause of liver dysfunction and failure after restoration of liver ischaemia, as occurs during liver transplantation [1], surgical resection [2] and trauma [3]. Prevention of IRI could be an important therapeutic strategy to prevent liver dysfunction, and improve the survival rate of patients who undergo liver operations.

Tumour necrosis factor (TNF)-α is a key inflammatory cytokine released by infiltrated leucocytes during IRI [4,5]. Inhibition of TNF-α can effectively prevent IRI, and may be a therapeutic target to prevent IRI [6–8]. Recent studies have revealed that TNF-α promotes hepatic IRI through an NF-κB-dependent pathway [8]. However, IRI is a multicomponent and multistep process involving cAMP-PKA [9]. The MAPK/ERK pathway [10] controls and regulates mitochondrial energy production, an abnormality of which can result in IRI. Hence, the pathways of TNF-α that involved in IRI need to be elucidated.

Energy metabolism in mammalian cells is dependent on continuous mitochondrial activity such as mitochondrial fusion, fission and mitochondrial remodelling [11]. Mitofusion2 (Mfn2) is an integral outer mitochondrial membrane protein that is essential for mitochondrial fusion [12,13]. Loss or down-regulation of Mfn2 expression can lead to disturbance of mitochondrial fusion and dysmetabolism of mitochondrial energy [12]. Peroxisome proliferator–activated receptor-γ co-activator (PGC)-1α is a thermogenic transcriptional co-activator that is involved in energy metabolism through regulation of mitochondrial biogenesis and respiration [12,14]. Loss of PGC-1α expression can result in defects of adenosine triphosphate (ATP) production in response to physiological stimuli [14]. PGC-1α has recently been confirmed to be involved in a mitochondrial regulatory pathway through interaction with Mfn2 in skeletal muscle cells and heart cells [15,16]. Consequently, PGC-1α/Mfn2 may constitute a cascade pathway that regulates mitochondrial energy metabolism which is impaired during IRI.

The aim of the study was to determine the role of the TNF-α/PGC-1α/Mfn2 pathway in the pathogenesis of liver IRI in a rat model of IRI.

## Materials and methods

Expanded materials and methods are available in the Data S1.

### Animals and human liver samples

Inbred male Sprahue-Dawley rats (200–250 g) and BALB/c mice (6 and 8 weeks old) were purchased from the Center of Experimental Animals (Tongji Medical College, Huazhong University of Science and Technology, China). Animal handling procedures were conducted in compliance with guidelines for the Care and Use of Laboratory Animals published by NIH, and all animal experimental protocols were approved by the Animal Care and Use Committee of Tongji Medical College. Human liver specimens were obtained from patients with partial liver resection in Union Hospital, Tongji Medical College of Huazhong University of Science and Technology. The experiments were carried out in accordance with the Declaration of Helsinki (2000) of the World Medical Association and the protocols approved by the Institutional Research Review Board at Union Hospital, Tongji Medical College of Huazhong University of Science and Technology with informed consent.

### Lentiviral vectors production

Recombinant lentiviral vectors carrying a specific albumin promoter and an Mfn2 (Ltv-Mfn2) gene downstream fused to enhanced green fluorescence protein (EGFP) were generated as described previously [17–19].

### Cell culture and treatment

L02 cell line was purchased from China Center for Type Culture Collection (Wuhan, China).

For drug treatment, L02 cells were first incubated by recombinant human TNF-α (1 ng/ml, Cell Signaling Technology, 8902, Beverly, MA, USA) with or without neutralizing anti-TNF-α (1 μg/ml, Cell Signaling Technology, 7321) for 24 hrs. Additional recombinant human TNF-α (1 ng/ml, Cell Signaling Technology, 8902) was administered to L02 cells after pre-treatment with rosiglitazone (10 μmol/l, Sigma-Aldrich, R2408, St. Louis, MO, USA) for 24 hrs or transfection with Ltv-human-Mfn2 for 72 hrs.

For transfection treatment, L02 cells were transfected with Ltv-human-vector or Ltv-human-Mfn2 in the presence of polybrene (5 μg/ml). Transfection efficiency was monitored by fluorescence microscopy, quantitative PCR and Western Blotting after 48 hrs after transfection (Fig. S1). Experiments were performed 72 hrs after transfection. Cells without any treatment served as controls.

### Rat liver IR model and animal treatment

Rat model of partial warm hepatic IRI was produced as described by Ji H *et al*. [20].

For drug treatment, rats received rosiglitazone (3 mg/kg/day, Sigma-Aldrich, R2408) by intraperitoneal injection 2 weeks before the IR operation, and 10 μg of rat neutralizing anti-TNF-α (5 μg/ml, Cell Signaling Technology, 11969) was infused as soon as blood reperfusion was established. For transfection treatment, rats were exposed to 1 ml of Opti-MEM I medium, containing Ltv-rat-Mfn2 or Ltv-rat-vector (2 × 10^7^ TU/ml) in the presence of polybrene (5 μg/ml, Sigma-Aldrich, 107689, MO) administered by intravenous injection 1 week before the IR operation. Transfection efficiency was monitored by fluorescence microscopy and quantitative PCR (Fig. S2). Rats were killed after 12 hrs of blood reperfusion. Rats without operation were served as controls.

### Electronic microscopy

Mitochondrial morphology in L02 cells and liver samples were observed by transmission electron microscope (FEI/Philips TCNAI G2, Eindhoven, The Netherlands).

### ATP detection

L02 cells, 1 × 10^6^ and 20 mg liver sample per group were lysed in lysis buffer from an ATP Assay Kit (Beyotime, S0026, Jiangsu, China). They were centrifuged at 12,000 g for 10 min., and the supernatants collected. ATP production was then analysed according to the manufacturer's instructions.

### Alanine transarninase (ALT) activity detection

Supernatants of L02 cells and plasma samples were analysed for ALT activity by using an ALT Assay Kit (Abcam, ab105134, Cambridge, MA, USA). Briefly, supernatants of 1 × 10^6^ cells, and plasma samples were collected and centrifuged at 1000 *g* for 10 min. ALT activity was then detected in the supernatants by a colorimetric assay according to the manufacturer's instructions.

### Reactive oxygen species (ROS) detection

L02 cells and liver samples were analysed for ROS content by using a Reactive Oxygen Species Assay Kit (KeyGen BioTECH, KGT010, Nanjing, China). Briefly, 1 × 10^6^ cells, and 1 g liver samples were collected, rinsed with cleaning liquid from the kit, and then centrifuged at 300 × g for 5 min. ROS content of the precipitations was then measured by fluorescence spectrophotometry according to the manufacturer's instructions.

### TNF-α detection by ELISA

Each plasma sample was analysed for TNF-α by ELISA using an ELISA kit (R&D Systems, RTA00, Minneapolis, MN, USA) according to the manufacturer's instructions.

### Cell apoptosis assay by flow cytometry

L02 cell apoptosis was quantitatively assessed by flow cytometry (BD LSR II, BD Biosciences, San Jose, CA, USA) after labelling by propidium (PI, 10 μg/ml) and allophycocyanin-labelled Annexin V (Annexin V-APC; Bender MedSystems, eBioscience, 88-8007, San Diego, CA, USA).

### Tissue apoptosis assay by TUNEL

Apoptosis in liver samples was detected by using an In Situ Cell Death Detection Kit (Roche Diagnostics, 12156792910, Branford, CT, USA) according to the manufacturer's instructions.

### Gene expression assay by QRT–PCR

RNA from L02 cells and liver samples per group was isolated by TRIzol™ Reagent (Invitrogen, 15596026, Carlsbad, CA, USA) according to the manufacturer's protocol. RNA levels were measured by reverse transcription–polymerase chain reaction by using iQ SYBR Green Supermix in an iCycler Real-Time PCR Detection System (Bio-Rad, München, Germany).

### Protein expression assay by Western Blotting

Western Blotting was used to measure the levels of TNF-α, PGC-1α and Mfn2 in each group of L02 cells, and liver samples. Optical density of the bands was quantified by using NIH Image J software.

### Statistical analysis

All data were presented as mean ± SEM. Six rodents per group were used in the experiments. After demonstration of homogeneity of variance with the Bartlett test, one-way anova followed by Student–Newman–Keuls test where appropriate, was used to evaluate the statistical significance. Values of *P* < 0.05 were considered statistically significant. Experiments were performed in triplicate.

## Results

### TNF-α promotes hepatocyte injury with mitochondrial abnormality

**Fig. 1:**
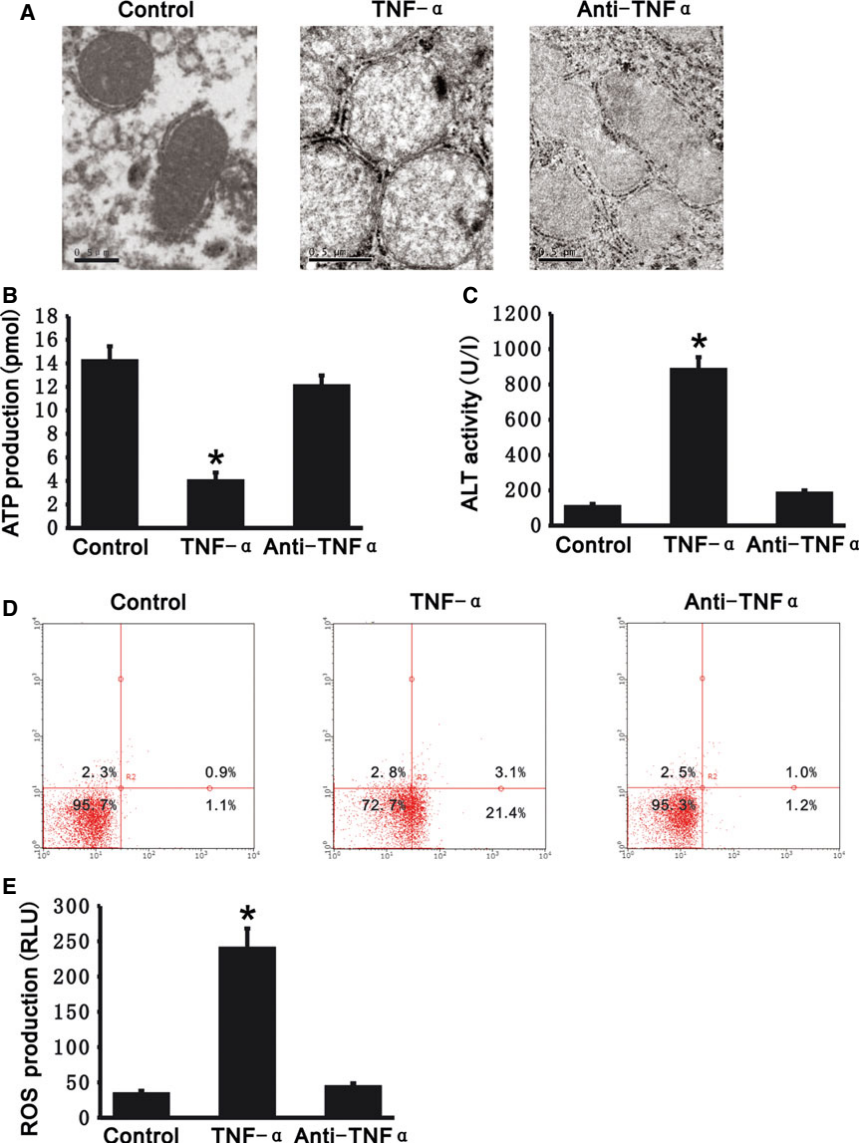
Tumour necrosis factor alpha (TNF-α)-induced hepatocellular injury. Treatment of exogenous recombinant TNF-α induced mitochondrial swelling, decreased mitochondria cristae, reduced adenosine triphosphate (ATP) production and promoted cell apoptosis as well as increased reactive oxygen species (ROS) production in L02 cells. However, the neutralizing anti-TNF-α antibody decreased the mitochondrial swelling, maintained the ATP production and protected cells from apoptosis as well as inhibited ROS production. (**A**) Mitochondrial morphology in L02 cells were observed by a transmission electron microscope; scale bar: 0.5 μm. (**B**) ATP production was measured in L02 cells with exogenous recombinant TNF-α or neutralizing anti-TNF-α. (**C**) ALT activity was measured in supernatants from L02 cells with exogenous recombinant TNF-α or neutralizing anti-TNF-α. (**D**) Quantitation of apoptotic cells was carried out by flow cytometry. Representative plots of annexin V-APC/PI flow cytometry from three independent experiments are presented. The number represents the percentage of cells in each quadrant. (**E**) ROS production was measured in L02 cells with exogenous recombinant TNF-α or neutralizing anti-TNF-α. All data presented are mean ± SEM (*n* = 5). *P < 0.05 *versus* control.

As depicted in Figure 1A, compared with control, mitochondria in L02 cells which were treated with exogenous recombinant human TNF-α appeared to be swollen, and the number mitochondria cristae decreased. ATP produced by mitochondria was also significantly reduced (3.97 ± 0.74 *versus* 14.17 ± 1.28 pmol) (Fig. 1B). ALT activity in the media of the L02 cells was increased (881.72 ± 72.71 *versus* 104.44 ± 18.38 U/l). Apoptosis in these cells as determined by PI and Annexin V labelling showed that there was more apoptosis in human TNF-α-treated *versus* control cells (24.5% *versus* 2.0%*)*, reflecting hepatocyte injury (Fig. 1C and D). However, neutralizing anti-TNF-α decreased the mitochondrial swelling, and maintained the ATP production (12.04 ± 0.93 pmol) in L02 cells treated with TNF-α. It also decreased the ALT activity in the media (180.54 ± 19.20 U/l), and decreased apoptosis (2.2%). We measured the production of ROS in L02 cells. TNF-α increased ROS production (239.22 ± 28.81 *versus* 32.94 ± 5.09 RLU), whereas neutralizing anti-TNF-α decreased it (43.04 ± 5.68 RLU) in L02 cells (Fig. 1E). These results indicate that TNF-α promoted hepatocyte injury by causing mitochondrial dysfunction.

### TNF-α inhibits the PGC-1α/Mfn2 pathway in hepatocytes

**Fig. 2:**
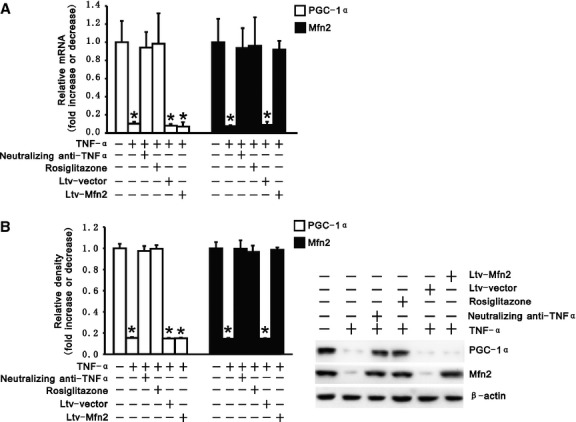
Tumour necrosis factor alpha (TNF-α) inhibited PGC-1α/Mfn2 pathway in hepatocytes. (**A**) mRNA expression of PGC-1α and Mfn2 was determined by quantitative RT-PCR in transfected and untransfected L02 cells as well as in L02 cells with neutralizing anti-TNF-α or rosiglitazone under treatment of exogenous recombinant TNF-α. mRNA levels are normalized to β-actin. (**B**) Protein levels of PGC-1α and Mfn2 was determined by Western Blots in transfected and untransfected L02 cells as well as in L02 cells with neutralizing anti-TNF-α or rosiglitazone under treatment of exogenous recombinant TNF-α. A representative Western Blot (right panel) of three independent experiments. Densitometric analyses (left panel) are presented as the relative ratio of each protein to β-actin. Data are shown as mean ± SEM (*n* = 5). *P < 0.05 *versus* untreated cells.

PGC-1α and Mfn2 play crucial roles in maintaining mitochondrial activity and function [12–14]. We explored whether TNF-α had an effect on PGC-1α and Mfn2 levels in hepatocytes. Real-time PCR and immunoblotting revealed that treatment of TNF-α in L02 cells inhibited expression of PGC-1α and Mfn2. However, compared with control, blockade of TNF-α by neutralizing antibody sustained expression of PGC-1α and Mfn2 (Fig. 2). Rosiglitazone, a peroxisome proliferator-activated receptor (PPAR)-γ agonist which has been shown to promote PGC-1α expression [21], was found to reverse the expression of PGC-1α in L02 cells treated with TNF-α. As shown in Figure 2, compared with cells treated by TNF-α, the decrease in PGC-1α expression also decreased the expression of Mfn-2 in L02 cells treated with TNF-α. Nevertheless, transfection of Ltv-Mfn2 did not reverse the expression of PGC-1α in the cells treated with TNF-α. These data suggest that PGC-1α and Mfn2 form a PGC-1α/Mfn2 cascade which is negatively regulated by TNF-α in hepatocytes.

### PGC-1α/Mfn2 pathway mediates TNF-α-induced hepatocyte injury with mitochondrial abnormality

**Fig. 3:**
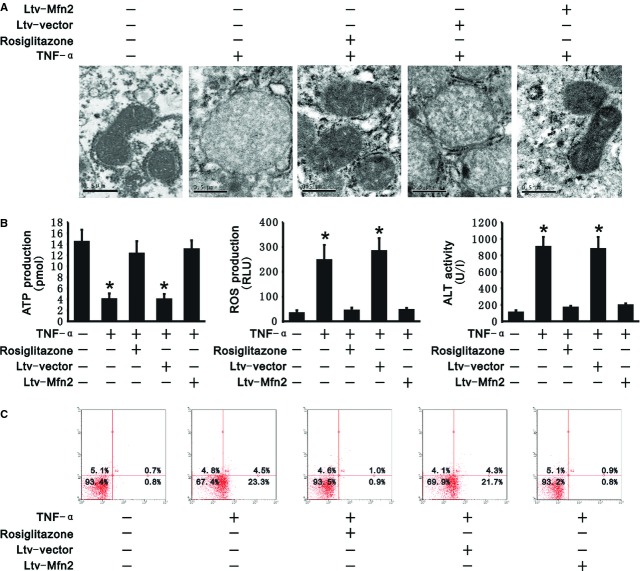
PGC-1α/Mfn2 pathway protected hepatocytes from tumour necrosis factor alpha (TNF-α)-induced injury. Rosiglitazone and transfection of Ltv-Mfn2 decreased mitochondrial swelling, maintained the adenosine triphosphate (ATP) production and protected cells from apoptosis as well as inhibited reactive oxygen species (ROS) production. (**A**) Mitochondrial morphology in L02 cells was observed by transmission electron microscopy; scale bar: 0.5 μm. (**B**) ATP (left panel) and ROS (middle panel) production was measured in transfected and untransfected L02 cells as well as in L02 cells with rosiglitazone under treatment of exogenous recombinant TNF-α. ALT activity (right panel) was measured in supernatants from transfected and untransfected L02 cells as well as from L02 cells with rosiglitazone under treatment of exogenous recombinant TNF-α. (**C**) Quantification of apoptotic cells was carried out by flow cytometry. Representative plots of annexin V-APC/PI flow cytometry from three independent experiments are presented. The number represents the percentage of cells in each quadrant. All data presented are mean ± SEM (*n* = 5). *P < 0.05 *versus* control.

We further explored the influence of PGC-1α/Mfn2 pathway on hepatocytes injury with TNF-α. Compared with L02 cells treated with TNF-α, both rosiglitazone and transfection of Ltv-Mfn2 decreased mitochondrial swelling and maintained ATP production (12.29 ± 2.27, 13.07 ± 1.62 pmol; Fig. 3A and B). Conversely, ROS production was significantly reduced by rosiglitazone and transfection of Ltv-Mfn2 (43.54 ± 11.76, 45.72 ± 8.74 RLU, respectively; Fig. 3B). As expected, reduction in mitochondrial swelling and ROS resulted in decreased ALT activity in the media of L02 cells treated with TNF-α (165.54 ± 23.22, 194.22 ± 21.93 U/l). Apoptosis of L02 cells as determined by PI and Annexin V labelling was also decreased (2.0%, 1.7%, respectively; Fig. 3B and C), indicating a protective role of rosiglitazone and Ltv-Mfn2 in mitochondria and hepatocytes against TNF-α. Since TNF-α inhibited whereas rosiglitazone and Ltv-Mfn2 increased PGC-1α/Mfn2 expression, we suggest that the PGC-1α/Mfn2 pathway plays a protective role in TNF-α-induced hepatocyte injury with mitochondrial dysfunction. Suppression of the PGC-1α/Mfn2 pathway mediates TNF-α-induced mitochondrial dysfunction and hepatocyte injury.

### Inhibition of TNF-α alleviates liver IRI

**Fig. 4:**
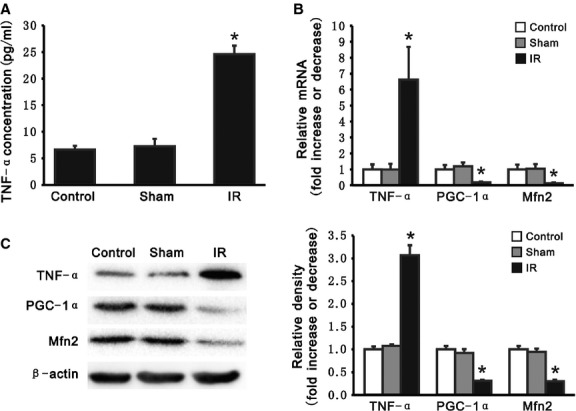
Tumour necrosis factor alpha (TNF-α) increased in rats with liver of IR. (**A**) Concentration (pg/ml) of TNF-α was measured by ELISA in plasma from rats with or without liver IR. (**B**) Levels of mRNA for TNF-α, PGC-1α and Mfn2 were determined by quantitative RT-PCR in livers from control, sham and IR groups. mRNA levels were normalized to β-actin. (**C**) Western Blot analysis demonstrated increased TNF-α, decreased PGC-1α and Mfn2 protein levels in livers from IR group, compared with those from control and sham groups. A representative Western Blot (left panel) of three independent experiments. Densitometric analyses (right panel) are presented as the relative ratio of each protein to β-actin. All data presented are mean ± SEM (*n* = 6 rats per group). *P < 0.05 *versus* control.

Compared with the control and sham operation groups, TNF-α concentrations were significantly increased in plasma from rats with liver IR. Real-time PCR as well as immunoblotting confirmed that TNF-α expression was up-regulated in rat livers with IR. Consistent with our results cell culture, expression of PGC-1α and Mfn2 were down-regulated by TNF-α expression (Fig. 4).

**Fig. 5:**
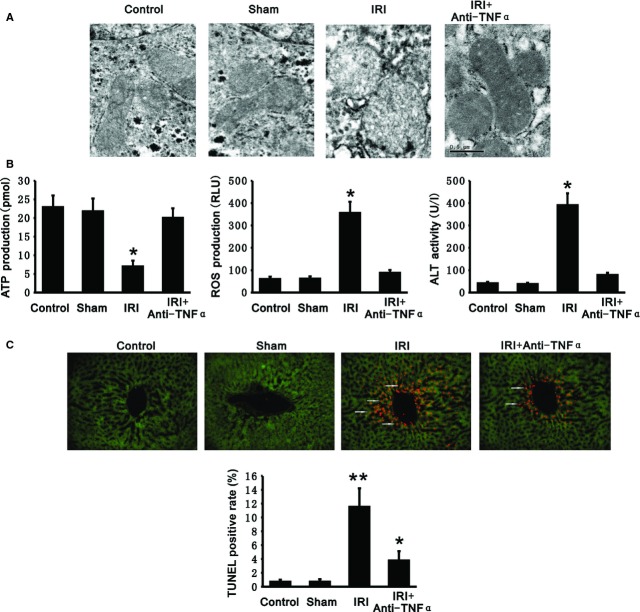
Inhibition of tumour necrosis factor alpha (TNF-α) alleviated liver ischaemia-reperfusion injury (IRI). Administration of neutralizing anti-TNF-α decreased mitochondrial swelling, maintained adenosine triphosphate (ATP) production, and protected hepatocytes from apoptosis as well as inhibited reactive oxygen species (ROS) production in livers with IR. (**A**) Mitochondrial morphology in hepatocytes was observed by transmission electron microscopy; scale bar: 0.5 μm. (**B**) ATP (left panel) and ROS production (middle panel) was measured in livers from control, sham and IR groups with or without neutralizing anti-TNF-α. ALT activity (right panel) was measured in livers from control, sham and IR groups with or without neutralizing anti-TNF-α. (**C**) Representative TUNEL-stained cross-sections from control, sham and IR groups with or without neutralizing anti-TNF-α showing apoptotic cells (upper panel). Arrowheads denote TUNEL positive cells. Cells were marked with green fluorescence by transfection of Ltv-vector-EGFP; scale bar: 20 μm. The bar graph (lower panel) shows the mean percentage of TUNEL positive hepatocytes (± SEM) from four livers for each experimental group in three sections. All data presented are mean ± SEM (*n* = 6 rats per group). **P < 0.01, *P < 0.05 *versus* control.

We further investigated the role of TNF-α in rat liver IRI by systemic administration of neutralizing anti-TNF-α. Comparing control and sham operation groups, mitochondria were swollen with disappearance of mitochondrial cristae and ATP production was decreased in livers with IR (7.04 ± 1.55 *versus* 22.92 ± 3.12 pmol; Fig. 5A and B). However, ROS production was elevated (355.79 ± 50.13 *versus* 60.35 ± 10.68 RLU). ALT activity in plasma from rat with liver IR was also significantly increased (390.60 ± 53.17 *versus* 41.22 ± 6.64 U/l; Fig. 5B), and TUNEL assays showed that hepatocytes displayed apparent increased apoptosis in livers with IR (11.5% *versus* 0.7%; Fig. 5C), indicating the presence of IRI in the livers. Nevertheless, systemic administration of neutralizing anti-TNF-α preserved the normal morphology of hepatocellular mitochondria, maintained ATP production (20.04 ± 2.55 pmol), and reduced ROS production (87.94 ± 13.26 RLU) in livers with IR (Fig. 5A and B). At the same time, inhibition of TNF-α by neutralizing antibody decreased ALT activity in plasma (78.44 ± 10.05 U/l) and decreased hepatocyte apoptosis in livers with IR (3.8%) (Fig. 5B and C). In brief, TNF-α appeared to mediate hepatic IRI, and inhibition of TNF-α appeared to decrease IRI of liver.

### Up-regulation of PGC-1α/Mfn2 pathway rescues TNF-α-induced liver IRI

**Fig. 6:**
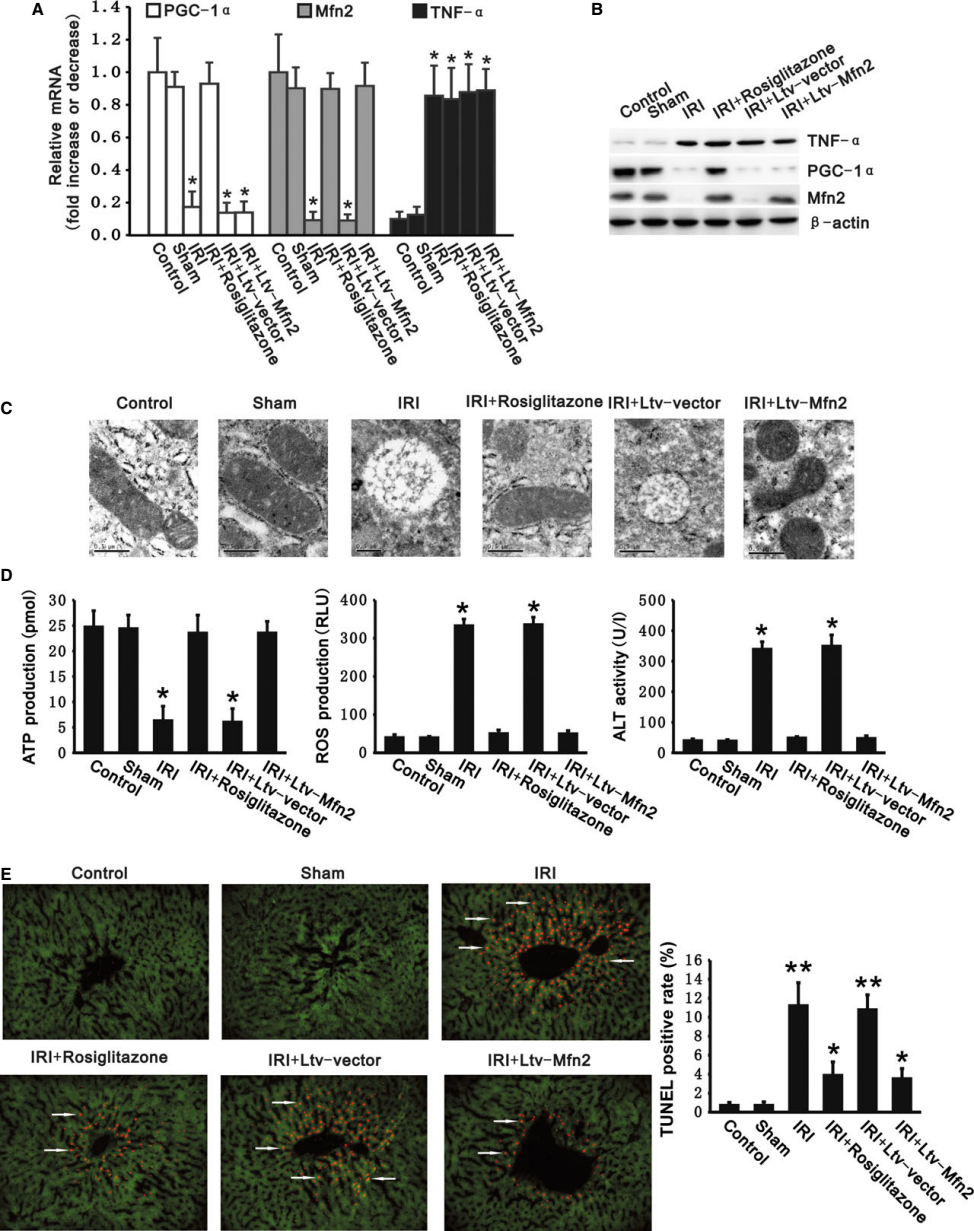
Up-regulation of PGC-1α/Mfn2 pathway rescued tumour necrosis factor alpha (TNF-α)-induced liver ischaemia-reperfusion injury (IRI). Rosiglitazone and transfection of Ltv-Mfn2 sustained expression of PGC-1α/Mfn2 pathway decreased mitochondrial swelling, maintained adenosine triphosphate (ATP) production, and protected hepatocytes from apoptosis as well as inhibited reactive oxygen species (ROS) production in livers with IR. (**A**) Expression of mRNA for PGC-1α, Mfn2 and TNF-α was determined by quantitative RT-PCR in livers from control, sham and IR groups with transfection or administration of rosiglitazone. mRNA levels were normalized to β-actin. (**B**) Representative Western Blots demonstrated PGC-1α, Mfn2 and TNF-α protein levels in livers from control, sham and IR groups with transfection or administration of rosiglitazone. (**C**) Mitochondrial morphology in hepatocytes was observed by transmission electron microscopy; scale bar: 0.5 μm. (**D**) ATP (left panel) and ROS (middle panel) production was measured in livers from control, sham and IR groups with transfection or administration of rosiglitazone. ALT activity (right panel) was measured in plasma from control, sham and IR groups with transfection or administration of rosiglitazone. (**E**) Representative TUNEL-stained cross-sections from control, sham and IR groups with transfection or administration of rosiglitazone showing apoptotic cells in livers (left panel). Arrowheads denote TUNEL positive cells. Cells were marked with green fluorescence by transfection of Ltv-vector-EGFP; scale bar: 20 μm. The bar graph (right panel) shows the mean percentage TUNEL positive hepatocytes (± SEM) from four livers for each experimental group in three sections. All data presented are mean ± SEM (*n* = 6 rats per group). **P < 0.01, *P < 0.05 *versus* control.

We confirmed PGC-1α/Mfn2 pathway was down-regulated in liver with IRI animals (Fig. 4). Rosiglitazone and transfection of Ltv-Mfn2 maintained PGC-1α/Mfn2 expression in hepatocytes treated with TNF-α cell culture (Fig. 2). Compared with the IR group, rosiglitazone activated the PGC-1α/Mfn2 pathway by up-regulating both PGC-1α and Mfn2 expression in livers with IR, whereas exposure to Ltv-Mfn2 alone resulted in overexpression of Mfn2 as the downstream site of activation of the PGC-1α/Mfn2 pathway. Neither rosiglitazone nor transfection with Ltv-Mfn2 decreased the overexpression of TNF-α in livers with IR (Fig. 6A, B and Fig. S3). These results suggest that rosiglitazone and Ltv-Mfn2 do not exert measurable feedback control on TNF-α.

In contrast to the IR group, up-regulation of the PGC-1α/Mfn2 pathway either by rosiglitazone or by transfection of Ltv-Mfn2 decreased mitochondrial swelling, and sustained ATP production (23.52 ± 3.56, 23.54 ± 2.29 pmol, respectively) with decreased ROS production (50.19 ± 9.74, 49.66 ± 8.70 RLU respectively) (Fig. 6C and D). The hepatocyte injury indicators, plasma ALT activity (48.35 ± 4.97, 46.73 ± 9.17 U/l respectively) and apoptosis of hepatocytes were both significantly diminished (3.9%, 3.5% respectively) (Fig. 6D and E). Briefly, rosiglitazone and Ltv-Mfn2 protected livers from IRI caused by TNF-α by activating the PGC-1α/Mfn2 pathway. Up-regulation of PGC-1α/Mfn2 pathway decreased TNF-α-induced liver IRI.

## Discussion

IRI increases the risk of death by causing liver dysfunction in patients with liver transplantation [1], surgical resection [2] and trauma [3]. IRI is a complex process with mitochondrial injury and involves multiple inflammatory factors participation. Many reports have described a critical role for TNF-α in the progression of IRI. However, relatively little is known regarding the precise contribution of TNF-α to the pathogenesis of liver IRI. In the current report, we provide the first direct evidence that PGC-1α and Mfn2 constitute a pathway that plays a central role in the development of TNF-α-induced liver IRI. *In vitro*, TNF-α induces mitochondrial injury, ATP production deficiency and increase in ROS that leads to hepatocyte injury and apoptosis by sequentially activating the PGC-1α/Mfn2 pathway. *In vivo*, rats which underwent IR operations had increased TNF-α and the down-regulation of the PGC-1α/Mfn2 pathway that results in hepatocyte injury, as was observed in the cell culture experiments. Furthermore, either inhibition of TNF-α or activation of PGC-1α/Mfn2 pathway by pharmacologic inhibitors or gene transfection decreased IRI in hepatocytes. These findings suggest that liver IRI induced by TNF-α is dependent on the PGC-1α/Mfn2 pathway.

Tumour necrosis factor-α is produced by a variety of inflammatory cells including T cells, B cells, NK cells and macrophages during various pathological and pathophysiological processes, such as tumour development [22–24], inflammation [22,25] and ischaemia-reperfusion [4,5,22]. TNF-α plays a key regulatory role both in cell survival, and apoptosis depending on the cell type and biological context [26]. TNF-α production has been shown to be increased in livers with IR during which inflammatory cells infiltrated and accumulated in target organs [27]. On the other hand, increases in TNF-α-induced hepatocyte injury and apoptosis both in cell culture and in animals during IR, have suggested that TNF-α promotes hepatocyte apoptosis under IR conditions.

Tumour necrosis factor-α has been implicated in the progression of IRI in variety organs, and inhibition of TNF-α has been shown to protect organs from IRI. These organs include heart [28], brain [29], kidney [30] and liver [6,8]. Recent studies have reported that TNF-α promotes hepatic IRI by regulating an NF-κB-dependent pathway [8]. However, IRI is a multicomponent and multistep involved pathophysiological procedure. cAMP-PKA and MARK/ERK pathway are also involved in regulation of IRI [9,10]. Hence, details of the role of TNF-α in the development of IRI of liver is still unclear. In this study, PGC-1α and Mfn2 were down-regulated in hepatocytes injured by TNF-α in IRI. We further investigated the role of PGC-1α and Mfn2 in TNF-α-induced hepatocyte injury by administration of a pharmacological agonist and gene transfection. This experimental model makes it possible to define the direct role of PGC-1α and Mfn2 in TNF-α-induced hepatocyte injury. In this model, maintenance of PGC-1α and Mfn2 expression by rosiglitazone or overexpression of Mfn2 by Ltv-Mfn2 transfection decreased mitochondrial swelling and hepatocyte injury even in presence of TNF-α in cell culture (Fig. 3) or with overexpression of TNF-α in animals (Fig. 6). Decreases in TNF-α by neutralizing antibody sustained the expression of PGC-1α and Mfn2. These findings indicate that TNF-α is an upstream factor for IRI of hepatocytes, and PGC-1α and Mfn2 play an active role in this process. Accordingly, inhibition of TNF-α or overexpression of PGC-1α and Mfn2 in hepatocytes represents a potential target for therapeutic intervention to prevent liver IRI.

Mitochondrial swelling, ATP production deficiency and excess ROS represent characteristics of IRI [31,32]. Mfn2 encodes an integral outer mitochondrial membrane protein that is essential for maintenance of the mitochondrial network, and that regulates mitochondrial metabolism, oxidative metabolism, and cell death *etc*. [12]. Loss or down-regulation of Mfn2 expression has been found in energy dysmetabolism-related diseases, such as obesity and diabetes [33,34]. In this study, mitochondrial swelling, ATP production deficiency, and increases in ROS resulted from the inhibition of Mfn2 expression by TNF-α, which led to hepatocyte injury and apoptosis. Overexpression of Mfn2 by Ltv-Mfn2 transfection maintained normal mitochondrial morphology, maintained ATP production, and reduced ROS, and thereby decreasing hepatocyte injury and apoptosis both in cell culture and in animals. These indicate that down-regulation of Mfn2 expression is also responsible for hepatic IRI. Interestingly, we also found that TNF-α inhibited PGC-1α expression and inhibition of PGC-1α expression resulted in similar mitochondrial and hepatocytic injury as had occurred in association with down-regulation of Mfn2. This result is in accordance with the concept that loss of PGC-1α interferes with energy metabolism and ATP production by dysregulating mitochondrial biogenesis and respiration [12,14]. These results suggest that PGC-1α is another contributor to hepatic IRI causing mitochondrial energy dysmetabolism.

PGC-1α has been documented to regulate activation of the MEK1/2 and NF-κB signalling pathway in muscle cells [35]. However, we found that PGC-1α regulated Mfn2 activation in hepatocytes. This is based on the observation that TNF-α inhibited both expression of PGC-1α and Mfn2, whereas the PPAR-γ agonist, rosiglitazone, increased PGC-1α and Mfn2 expression in hepatocytes. However, the overexpression of Mfn2 by Ltv-Mfn2 transfection did not reverse the TNF-α-induced low expression of PGC-1α. These data indicate that PGC-1α plays a role at an upstream site in stimulating Mfn2 expression in hepatocytes. The results are in accordance with a previous report that PGC-1α exerts a stimulatory effect on Mfn2 expression in muscle cells [15]. Another important finding of the current study is that PGC-1α and Mfn2 constitute a cascade pathway to regulate mitochondrial morphology and function in hepatocytes during IRI. The overexpression of Mfn2 by Ltv-Mfn2 transfection decreased TNF-α-induced mitochondrial dysfunction, ROS, hepatocyte injury and apoptosis even though PGC-1α expression was low. These data also suggest that Mfn2 is a downstream site of PGC-1α in hepatic mitochondrial regulation which is consistent with the report that they constitute a pathway to control mitochondrial action in muscle cells [15].

Other studies have reported that the PGC-1α/Mfn2 regulatory pathway is functional in diseases of energy dysmetabolism, such as type 2 diabetes which support our finding that PGC-1α/Mfn2 plays a key role in the regulation of hepatic mitochondrial function whose abnormality is involved in the pathogenesis of hepatic IRI, [36].

In summary, the findings reported here indicate that the PGC-1α/Mfn2 pathway plays a crucial role in the TNF-α-mediated hepatic IRI. Targeted manipulation of TNF-α and PGC-1α/Mfn2 pathway might protect hepatocytes from IRI.

## Supporting information

**Figure S1** The expression of target genes in transfected cells.

**Figure S2** The expression of target genes in livers.

**Figure S3** Densitometric analysis of Western blots for Figure 6B.

**Data S1** Expand Materials and Methods.
