# Supplementary material for: Tumour necrosis factor-α promotes liver ischaemia-reperfusion injury through the PGC-1α/Mfn2 pathway
Source: J Cell Mol Med. 2014 Jun 4;18(9):1863–73. doi: 10.1111/jcmm.12320 (PMC4196661; doi:10.1111/jcmm.12320)
Supplement: Supplementary file 1 — Figure S1 The expression of target genes in transfected cells. [file jcmm0018-1863-SD1.doc]

**Tumor necrosis factor-α Promotes Liver Ischemia-Reperfusion Injury through the PGC-1α/Mfn2 Pathway**

**Jun Li^1^, Wenbo Ke^2^, Qi Zhou^3^, Yongzhong Wu^4^, Hong Luo^1^, Hong Zhou^1^, Bin Yang^2^, Yu Guo^5^, Qichang Zheng^2^, Yong Zhang^2*^**

**Supplemental Materials**

**Expand Materials and Methods**

***Animals and human liver samples***

Inbred male Sprague-Dawley rats (200 to 250 g) and BALB/c mice (6 and 8 weeks old) were purchased from the Center of Experimental Animals (Tongji Medical College, Huazhong University of Science and Technology, China). Animal-handling procedures were conducted in compliance with guidelines for the Care and Use of Laboratory Animals published by NIH, and all animal experimental protocols were approved by the Animal Care and Use Committee of Tongji Medical College. Human liver specimens were obtained from patients with partial liver resection in Union Hospital, Tongji Medical College of Huazhong University of Science and Technology. The experiments were carried out in accordance with the Declaration of Helsinki (2000) of the World Medical Association and the protocols approved by the Institutional Research Review Board at Union Hospital, Tongji Medical College of Huazhong University of Science and Technology with informed consent.

***Lentiviral vectors production***

Recombinant lentiviral vectors carrying a specific albumin promoter and an Mfn2 (Ltv-Mfn2) gene downstream fused to enhanced green fluorescence protein (EGFP) were generated as described previously [1-3]. Briefly, the mouse albumin promoter (–449 to +100, GenBank accession no.NM_009654) from liver of Balb/c mouse and the human albumin promoter (–651 to +299, GenBank accession no.U31482) from human liver specimens were amplified using the polymerase chain reaction (PCR), and cloned into respective pMD19-T vector. Using a gene recombinant method, the mouse and human albumin promoters was separately cloned into the PacI and BamHI restriction sites, respectively of the lentiviral vector, pGC-LV, whose ubiquitin promoter was replaced by the respective albumin promoters. Subsequently, the rat and human Mfn2 gene (GenBank accession no.NM_130894 and no.NM_014874) was respectively inserted into the lentiviral vectors, resulting in the target gene fused to EGFP. The fusion gene was expressed under the promotion of specific albumin promoter. The recombinant lentiviral vector was co-transfected with packaging plasmids pHelper 1.0 and pHelper 2.0 into 293T cells growing in suspension using Lipofectamine 2000. Affinity chromatography was used to concentrate and purify lentiviral particles. Functional viral titers were determined using real-time PCR, RT-PCR).

***Cell culture and treatment***

The human hepatocyte cell line, L02 cell, was purchased from China Center for Type Culture Collection (Wuhan, China). Cell lines were cultured in RPMI 1640 medium supplemented with 10% fetal bovine serum (Hyclone, SV30087.01, Boston, MA) and 100 μg/ml each of penicillin and streptomycin (Gibco, Invitrogen, 15140-122, Carlsbad, CA) in 5% CO_2_ at 37°C.

To examine the influence of TNF-α on PGC-1α/Mfn2 pathway, and the subsequent biological effect in hepatocytes, L02 cells were first incubated by recombinant human TNF-α (1 ng/ml, Cell Signaling Technology, 8902, Beverly, MA) with or without neutralizing anti-TNF-α (1 μg/ml, Cell Signaling Technology, 7321) for 24 h. Additional recombinant human TNF-α (1 ng/ml, Cell Signaling Technology, 8902) was administered to L02 cells after pretreatment with rosiglitazone (10 μmol/L, Sigma-Aldrich, R2408, MO) for 24 h or transfection with Ltv-human-Mfn2 for 72 h.

For transfection treatment, twenty-four h-prior to transfection, L02 cells were plated onto 6 well plates (2×10^4^ cells/well) and incubated for 12 h with Opti-MEM I medium (Gibco, Invitrogen, 31985, Carlsbad, CA) containing a 200 multiplicity of transfection (MOI) per cell of Ltv-human-vector or Ltv-human-Mfn2 in the presence of polybrene (5 μg/ml), after which the Opti-MEM I medium was replaced with complete medium. Transfection efficiency, monitored using EGFP expression (fluorescence microscope, Olympus, Japan), was greater than 80% (Supplemental Figure IA). The overexpression of Mfn2 in transfected L02 cells was confirmed by quantitative PCR and Western Blotting after 48 h after transfection (Supplemental Figure IB and IC). Experiments were performed 72 h after transfection. Cells without any treatment served as control.

***Rat liver IR model and animal treatment***

Rat model of partial warm hepatic IRI was produced as described by Haofeng et al [4]. In brief, the hepatic arterial and portal venous blood supply to the central and left lobes was interrupted by atraumatic clips for 90 min to generate ischemia. Control rats had a sham operation by the same procedure, but without interruption of the blood supply.

For drug treatment, rats received rosiglitazone (3 mg/kg/d, Sigma-Aldrich, R2408) by intraperitoneal injection 2 weeks before the IR operation, and 10 μg of rat neutralizing anti-TNF-α (5 μg/ml, Cell Signaling Technology, 11969) was infused as soon as blood reperfusion was established. For transfection treatment, rats were exposed to 1 ml of Opti-MEM I medium, containing Ltv-rat-Mfn2 or Ltv-rat-vector (2×10^7^TU/ml) in the presence of polybrene (5μg/ml, Sigma-Aldrich, 107689, MO) administered by intravenous injection 1 week before the IR operation. Transfection efficiency was monitored by fluorescence microscopy and quantitative PCR (Supplemental Figure II). Rats were sacrificed after 12 h of blood reperfusion. Liver and serum samples were collected for analysis. Rats without operation were served as controls.

***Electronic microscopy***

L02 cells were collected at density of 5×10^6^. After rinsing in PBS, cells were centrifuged at 4000 r/min for 15 min and liver samples were cut into 1 mm^3^ sections at 4^o^C. They were then fixed in 2.5% glutaraldehyde for 1 h. Following rinsing in PBS to remove the glutaraldehyde, cells and liver samples were refixed in 1% osmic acid solution for 1 h. Subsequently, they were dehydrated in sequential gradients of ethanol and then embedded in mixture of acetone and epoxy resins for 2 h. After incubation in embedding medium overnight, they were dried and sliced into ultrathin sections, 50 nm. Finally, sections were stained by lead acetate-uranium for 10 min and observed under a transmission electron microscope（**FEI/Philips TCNAI G2, Netherlands）.**

***Adenosine triphosphate (ATP) detection***

L02 cells, 1×10^6^ and 20 mg liver sample per group were lysed in lysis buffer from an ATP Assay Kit (Beyotime, S0026, Jiangsu, China). They were centrifuged at 12000 g for 10 min, and the supernatants collected. ATP production was analyzed by using ATP Assay Kit (Beyotime) according to the manufacturer’s instructions.

***Alanine transarninase (ALT) activity detection***

Supernatants of L02 cells and plasma samples were analyzed for ALT activity by using an ALT Assay Kit (Abcam, ab105134, Cambridge, MA). Briefly, supernatants of 1×10^6^ cells, and plasma samples were collected and centrifuged at 3,000 rpm for 10 min. ALT activity was then detected in the supernatants by a colorimetric assay according to the manufacturer’s instructions.

***Reactive oxygen species (ROS) detection***

L02 cells and liver sample per group were analyzed for ROS content by using a Reactive Oxygen Species Assay Kit (KeyGen BioTECH, KGT010, Nanjing, China). Briefly, 1×10^6^ cells, and 1 g liver samples were collected, rinsed with cleaning liquid from the kit, and then centrifuged at 300 X g for 5 min. ROS content of the precipitations was then measured by fluorescence spectrophotometry according to the manufacturer’s instructions.

***TNF-α detection by ELISA***

Each plasma sample was analyzed for TNF-α by enzyme-linked immunosorbent assay (ELISA) using an ELISA kit (R&D Systems, RTA00, Minneapolis, MN) according to the manufacturer’s instructions.

***Cell apoptosis assay by flow cytometry***

L02 cell apoptosis was quantitatively assessed by flow cytometry. L02 cells were harvested, washed, and incubated with binding buffer containing propidium (PI, 10 μg/ml) and allophycocyanin-labeled Annexin V (Annexin V-APC) (Bender MedSystems, eBioscience, 88-8007, San Diego, CA) for 15 min at room temperature. Cell apoptosis was analyzed by flow cytometry (BD LSR II, BD Biosciences, San Jose, CA).

***Tissue apoptosis assay by TUNEL***

Apoptosis in liver samples was detected using an In Situ Cell Death Detection Kit (Roche Diagnostics, 12156792910) according to the manufacturer's instructions. Briefly, paraffin sections were deparaffinized and rehydrated through graded solutions of ethanol/water. Sections were treated for 15 min with 20 μg/ml proteinase K in PBS at 37°C, followed by incubation for 90 min at 37°C with terminal deoxynucleotidyl transferase (TdT) buffer containing deoxynucleotidyl transferase and biotinylated dUTP. For quantitative comparison of apoptotic cells, the percentage was calculated as the number of TUNEL-positive cells/total number of cells.

***Gene expression assay by QRT–PCR***

RNA from L02 cells and liver samples per group was isolated by TRIzol™ Reagent (Invitrogen, 15596026) according to the manufacturer’s protocol. Total RNA was reverse transcribed into first-strand cDNA using an iScript cDNA Synthesis kit (Bio-Rad, 170-8891, München, Germany). RNA levels were measured by reverse transcription–polymerase chain reaction using iQ SYBR Green Supermix in an iCycler Real-Time PCR Detection System (Bio-Rad). The following primer sequences were used—human PGC-1α sense: 5’-AAGGTCTCCAGGCAGTAG-3’, antisense: 5’-CACAGGTATAACGGTAGGTAA-3’; human Mfn2 sense: 5’-AGAGGCGTAAGGAGTAGG-3’, antisense: 5’-GAGGTTGGCTATTGATTGAC-3’; rat TNF-α sense: 5’-CGTGTTCATCCGTTCTCTA-3’, antisense: 5’-ATCTTCAGCAGCCTTGTG-3’; rat PGC-1α sense: 5’-CCTCCATGCCTGACGGCACC-3’, antisense: 5’-GAGCTGAGTGTTGGCTGGCG-3’; rat Mfn2 sense: 5’-TGGGATGCGTCTGCCTCGGA-3’, antisense: 5’-GAAGATCCGGTCCCCGGCCT-3’. Expression was normalized to that of β-actin.

***Protein expression assay by Western Blotting***

Western Blotting was used to measure the levels of TNF-α, PGC-1α and Mfn2 in each group of L02 cells and liver samples. Cells and liver samples were lysed in radioimmunoprecipitation assay (RIPA) buffer supplemented with a protease inhibitor cocktail (Roche, Branford, CT) and phosphatase inhibitor cocktail (Cell Signaling Technology, 5870). Total protein (30 μg) from each sample was electrophoresed on 12% sodium dodecyl sulfate–polyacrylamide gel electrophoresis gels. After transfer to nitrocellulose membranes (Pierce, Thermo Fisher Scientifc, 77012, Waltham, MA), protein samples were incubated with the corresponding human or rat primary antibodies (1:1000) (Abcam, Cambridge, MA). Blots were incubated with the appropriate horseradish peroxidase-conjugated secondary antibodies, and the membranes were developed with SuperSignal™ chemiluminescence reagent (Pierce, Thermo Fisher Scientifc, 34075) according to the manufacturer’s protocol. Protein expression levels were normalized against β-actin. Optical density of the bands was quantified using the NIH Image J software.

***Statistical analysis***

All data were presented as mean ± SEM. Six rodents per group were used in the experiments. After demonstration of homogeneity of variance with the Bartlett test, one-way analysis of variance followed by Student–Newman–Keuls test where appropriate, was used to evaluate the statistical significance. Values of P < 0.05 were considered statistically significant. Experiments were performed in triplicate.

**Supplemental Figure I:** *The expression of target genes in transfected cells.* (A) Recombinant lentiviral vectors were generated carrying a speciﬁc albumin promoter and the downstream EGFP with or without Mfn2 gene. Lentiviral particles were prepared and used to transfect L02 cells. Fluorescence microscopy of the transfected cells was performed 48 h after transfection. Transfection efﬁciency was greater than 90%. Original magniﬁcation: ×40. (B) mRNA expression of Mfn2 was determined by quantitative RT-PCR in transfected and untransfected L02 cells. mRNA levels were normalized to β-actin. (C) Western Blot analysis showed increased Mfn2 protein levels in Ltv-Mfn2 transfected L02 cells compared with untransfected L02 cells. Representative Western Blot (left panel) of three independent experiments. Densitometric analyses (right panel) are presented as the relative ratio of each protein to β-actin. Data are shown as mean ± SEM (n=4). ∗p<0.05 versus untransfected L02 cells.

**Supplemental FigureⅡ:** *The expression of target genes in livers.* (A) Livers were harvested from rats after one week after transfection. Frozen sections were prepared and assessed by ﬂuorescence microscopy. GFP expression conﬁned to hepatocytes is noted in sections from Ltv-vector and Ltv-Mfn2 transfected livers. Scale bar: 100 μm. (B) mRNA expression of Mfn2 was determined by quantitative RT-PCR in livers from rats after one week transfection. mRNA levels were normalized to β-actin. Data are shown as mean ± SEM (n=6 rats per group). ∗p<0.05 versus untransfected livers.

**Supplemental Figure III:** *Densitometric analysis of Western blots for Figure 6B.* Densitometric analyses of PGC-1α, Mfn2 and TNF-α normalized to β-actin in livers from control, sham and IR groups with transfection or administration of rosiglitazone. Data are representative of 3 independent experiments and shown as mean ± SEM (n=6 rats per group). ∗p<0.05 versus control.

**Supplemental References**

1. **Tiscornia G, Singer O, Verma IM.** Production and purification of lentiviral vectors. *Nat Protoc*. 2006; 1: 241-5.

2. **Sena-Esteves M, Tebbets JC, Steffens S, *et al.*** Optimized large-scale production of high titer lentivirus vector pseudotypes. *J Virol Methods*. 2004; 122: 131-9.

3. **Li J, Liu S, Li W, et al.** Vascular smooth muscle cell apoptosis promotes transplant arteriosclerosis through inducing the production of SDF-1alpha. *Am J Transplant*. 2012; 12: 2029-43.

4. **Ji H, Shen XD, Zhang Y, et al.** Activation of cyclic adenosine monophosphate-dependent protein kinase a signaling prevents liver ischemia/reperfusion injury in mice. *Liver Transpl*. 2012; 18: 659-70.
