# Supplementary figures and images for: Tumour necrosis factor-α promotes liver ischaemia-reperfusion injury through the PGC-1α/Mfn2 pathway
Source: J Cell Mol Med. 2014 Jun 4;18(9):1863–73. doi: 10.1111/jcmm.12320 (PMC4196661; doi:10.1111/jcmm.12320)

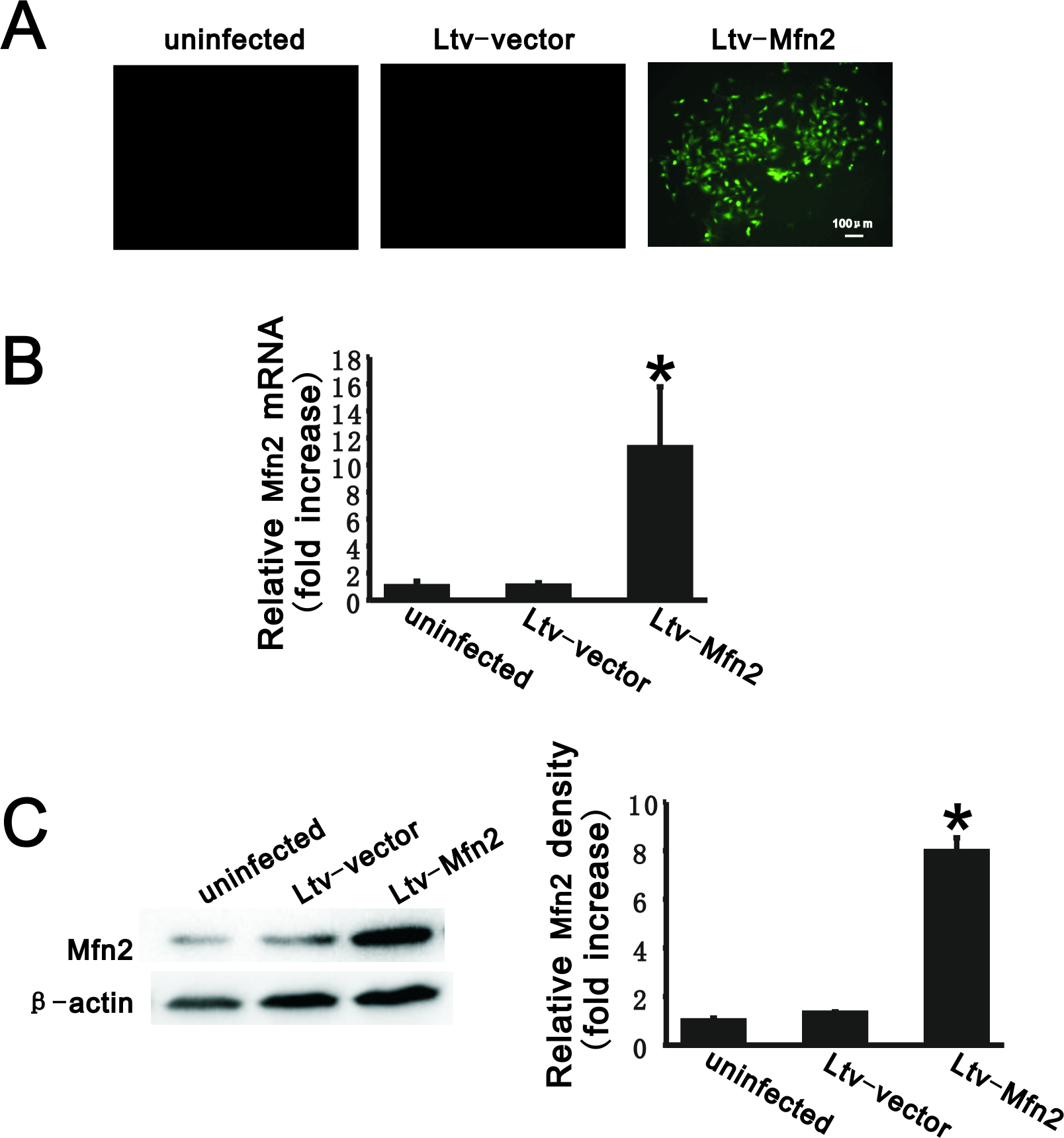

Supplement: Supplementary file 2 — Figure S2 The expression of target genes in livers. [file jcmm0018-1863-SD2.tif]

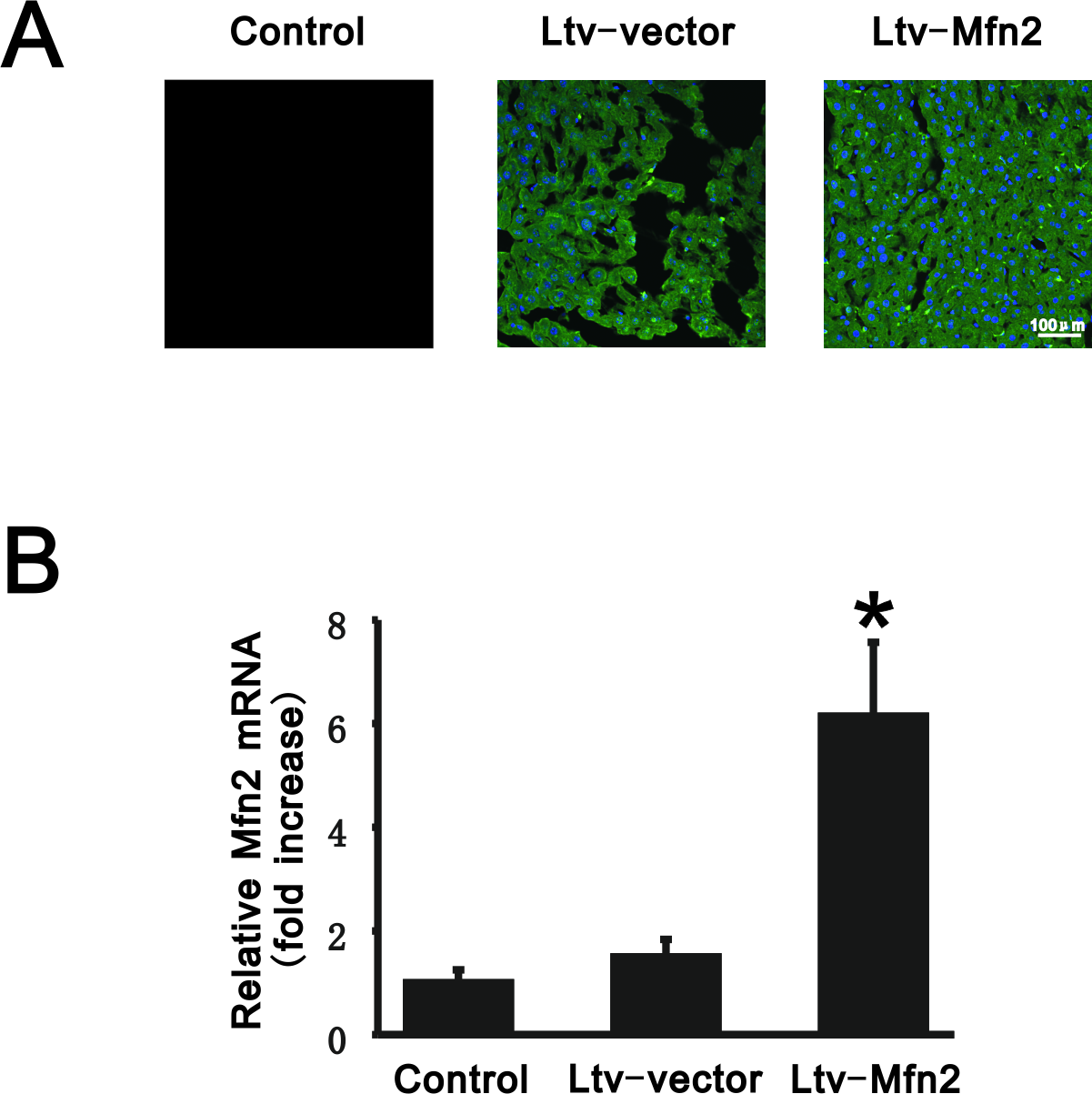

Supplement: Supplementary file 3 — Figure S3 Densitometric analysis of Western blots for Figure 6B. [file jcmm0018-1863-SD3.tif]

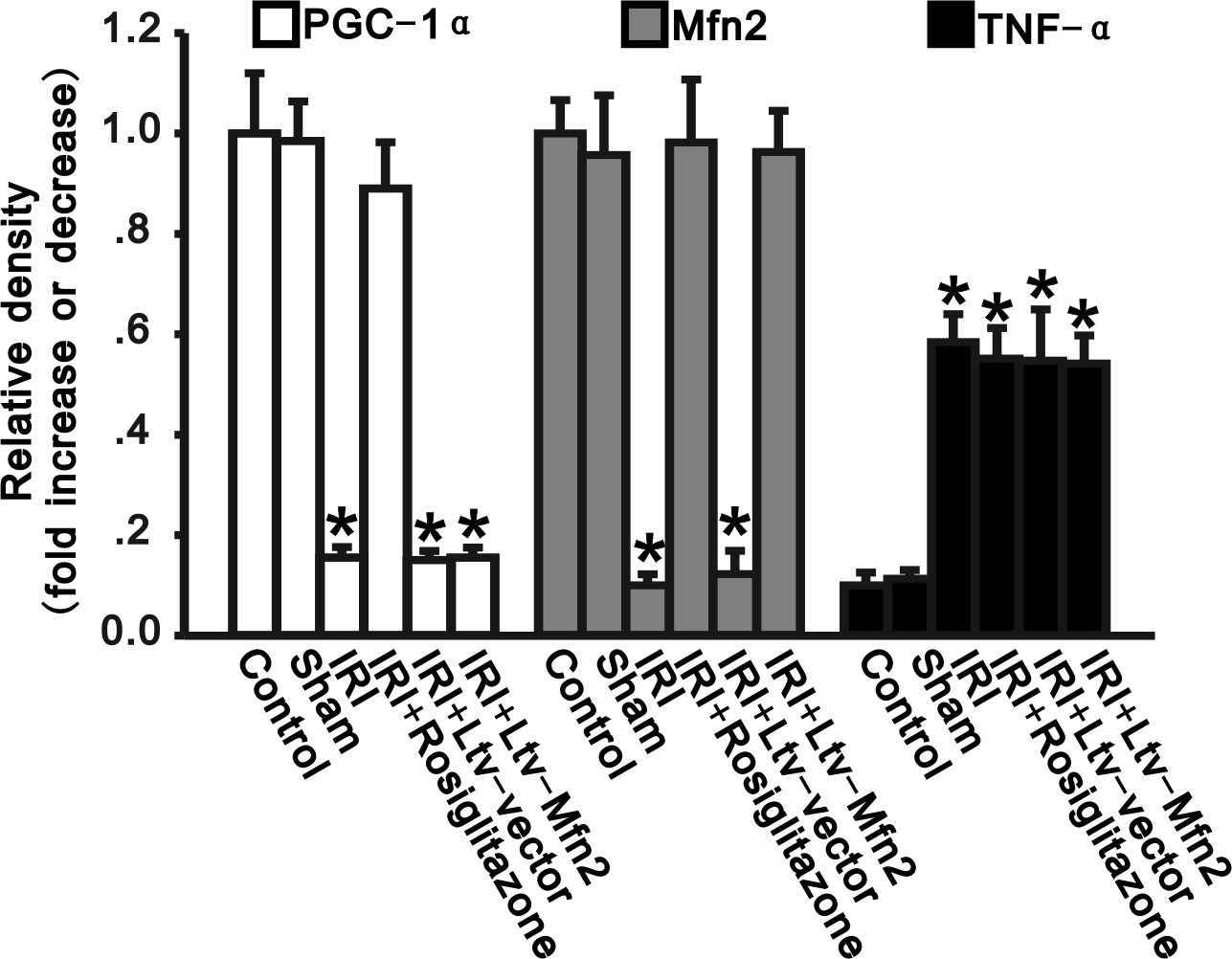

Supplement: Supplementary file 4 — Data S1 Expand Materials and Methods. [file jcmm0018-1863-SD4.tif]
